# Supportive Self-Management Program for People With Chronic Headaches and Migraine: A Randomized Controlled Trial and Economic Evaluation

**DOI:** 10.1212/WNL.0000000000201518

**Published:** 2023-03-28

**Authors:** Martin Underwood, Felix Achana, Dawn Carnes, Sandra Eldridge, David R. Ellard, Frances Griffiths, Kirstie Haywood, Siew Wan Hee, Helen Higgins, Dipesh Mistry, Hema Mistry, Sian Newton, Vivien Nichols, Chloe Norman, Emma Padfield, Shilpa Patel, Stavros Petrou, Tamar Pincus, Rachel Potter, Harbinder Sandhu, Kimberley Stewart, Stephanie J.C. Taylor, Manjit S. Matharu

**Affiliations:** From the Warwick Clinical Trials Unit (M.U., F.A., D.E., H.H., D.M., H.M., V.N., C.N., E.P., S.P., R.P., H.S., K.S.), and Division of Health Sciences (F.G., K.H., S.W.H.), Warwick Medical School University of Warwick, Coventry; University Hospitals Coventry and Warwickshire (M.U., D.E., H.M.), Coventry; University College of Osteopathy (D.C.), London; Wolfson Institute of Population Health (S.E., S.N., S.J.C.T.), Barts and the London School of Medicine and Dentistry, Queen Mary University of London; Nuffield Department of Primary Care Health Sciences (S.P.), University of Oxford; Department of Psychology (T.P.), Royal Holloway University of London; and Headache Group Institute of Neurology (M.S.M.) and the National Hospital for Neurology and Neurosurgery, Queen Square, London, United Kingdom.

## Abstract

**Background and Objectives:**

Chronic headache disorders are a major cause of pain and disability. Education and supportive self-management approaches could reduce the burden of headache disability. We tested the effectiveness of a group educational and supportive self-management program for people living with chronic headaches.

**Methods:**

This was a pragmatic randomized controlled trial. Participants were aged 18 years or older with chronic migraine or chronic tension–type headache, with or without medication overuse headache. We primarily recruited from general practices. Participants were assigned to either a 2-day group education and self-management program, a one-to-one nurse interview, and telephone support or to usual care plus relaxation material. The primary outcome was headache related-quality of life using the Headache Impact Test (HIT)-6 at 12 months. The primary analysis used intention-to-treat principles for participants with migraine and both baseline and 12-month HIT-6 data.

**Results:**

Between April 2017 and March 2019, we randomized 736 participants. Because only 9 participants just had tension-type headache, our main analyses were on the 727 participants with migraine. Of them, 376 were allocated to the self-management intervention and 351 to usual care. Data from 586 (81%) participants were analyzed for primary outcome. There was no between-group difference in HIT-6 (adjusted mean difference = −0.3, 95% CI −1.23 to 0.67) or headache days (0.9, 95% CI −0.29 to 2.05) at 12 months. The Chronic Headache Education and Self-management Study intervention generated incremental adjusted costs of £268 (95% CI, £176–£377) (USD383 [95% CI USD252–USD539]) and incremental adjusted quality-adjusted life years (QALYs) of 0.031 (95% CI −0.005 to 0.063). The incremental cost-effectiveness ratio was £8,617 (USD12,322) per QALY gained.

**Discussion:**

These findings conclusively show a lack of benefit for quality of life or monthly headache days from a brief group education and supportive self-management program for people living with chronic migraine or chronic tension–type headache with episodic migraine.

**Trial Registration Information:**

Registered on the International Standard Randomized Controlled Trial Number registry, ISRCTN79708100 16th December 2015 doi.org/10.1186/ISRCTN79708100. The first enrollment was April 24, 2017.

**Classification of Evidence:**

This study provides Class III evidence that a brief group education and self-management program does not increase the probability of improvement in headache-related quality of life in people with chronic migraine.

Globally, headaches are second only to back pain as a cause of years lived with disability.^1^ For epidemiologic purposes, chronic headache can be defined as having a headache for 15 or more days per month for at least 3 months.^2^ Approximately 3% of the worldwide population has such headaches.^3^ Most of these are due to chronic migraine or chronic tension–type headache with, or without, episodic migraine.^1^ Many also have medication overuse headache.^4-7^ Undiagnosed migraine is common in people with chronic headache disorders.^7^ Appropriate use of specific migraine treatments and analgesics may improve outcomes for people living with chronic headache disorders. Multiple psychosocial factors including anxiety, depression, poor sleep, medication overuse, and low self-efficacy for managing headaches are predictive of poor prognosis for people with chronic headaches.^8^ A 2017 systematic review (16 trials, n = 1811) including people with a mixture of headache types found small statistically significant benefits for nonpharmacological self-management on pain intensity, headache-related disability, quality of life, and mood, but, no effect on headache frequency.^9^ A 2019 Cochrane review of psychological interventions for migraine (21 trials, n = 2,482) did not find positive effects on migraine frequency or migraine-related disability.^10^ Supportive self-management approaches are effective for several chronic pain syndromes, but there is little evidence around people with chronic headache disorders.^11-13^ This randomized controlled trial tested the effect of a group educational and supportive self-management program on headache-related quality of life for people living with chronic headaches.

## Methods

### Study Design

The Chronic Headache Education and Self-management Study (CHESS) was a randomized controlled trial conducted in 2 localities in the United Kingdom: Greater London and the Midlands. The protocol for this trial has been published.^14^

### Study Population

We primarily recruited from general practices, but people could self-refer. Participating general practices ran computer searches to identify people who had, in the previous 2 years, consulted with headaches or who had been given a prescription for a migraine-specific drug (triptans/pizotifen). After screening for those whom it would be inappropriate to approach, practices sent letters, with 1 reminder, inviting people to find out more about the trial. The study team contacted interested responders by phone to confirm eligibility and posted baseline questionnaires, paper or electronic headache diary instructions, and consent forms. When these were returned, we arranged a headache classification telephone interview with a research nurse. To exclude people with ineligible headache types requiring specific treatment, for example, cluster headaches, and to describe our study population, we used a previously validated headache classification interview.^15^ Those with an ineligible headache type had a second telephone interview with a doctor from the National Migraine Center^16^ to confirm the diagnosis and, if appropriate, we directed them to their general practitioner for treatment. Eligible participants were not informed of their classification interview results until after randomization.

Our population of interest were adults meeting an epidemiologic definition of chronic headaches (≥15 headache days per month for at least 3 months) with migraine or tension-type headaches. For reporting, we identified 3 phenotypes, people with:

1. ICHD-3 criteria for chronic migraine; that is, at least 8 days per month with a migraine attack with or without aura,
2. less than 8 migraine attacks per month, or any number of attacks meeting ICHD-3 criteria for episodic migraine and chronic tension–type headache, and
3. chronic tension–type headache.^17^ In each group, we included those with and without medication overuse headache.

The target population for this trial was people managed in primary care, many of whom do not have a formal headache diagnosis. Diagnostic advice was part of the intervention. This fits the point in the care pathway at which this intervention would be delivered. In this study, we report on these groups together reflecting the information needed by those who might want to commission this service in primary care. We excluded those unable to attend the group self-management sessions, without access to a telephone, not fluent in English, or unable to participate in the group intervention for health reasons.

Our original objective was to test the effectiveness of an education and self-management support program for people meeting the epidemiologic definition of chronic headaches, with its effect on people with chronic migraine and those with chronic tension–type headache and episodic migraine as a secondary analysis. However, our feasibility study found that 95% of those recruited had either episodic or chronic migraine.^15,18^ With the agreement of the funder, trial steering committee, and data monitoring committee, we specified that if ≤15% of participants had chronic tension–type headache only, our primary analysis would be for those with migraine (with or without medication overuse headache), and the overall effect would be a secondary analysis.

### Randomization and Masking

We used block minimization to randomize individual participants in batches of approximately 20 to ensure we could populate the self-management groups in a timely manner. We stratified by geographical locality (Midlands and Greater London) and 6 headache types (chronic migraine, chronic tension–type headache and episodic migraine, and chronic tension–type headache; each with or without medication overuse headache). The randomization program was written specifically for this trial by Warwick Clinical Trials Unit programming team. The algorithm minimized the imbalance between the 2 trial arms using the stratifying groups and ensuring the allocation ratio fidelity. Randomization was performed by a member of staff independent of the CHESS research team. We maintained strict allocation concealment and all baseline data were collected prior to randomization. It was not possible to mask the study team and participants from the treatment allocation.

### Intervention

Our intervention development process has been published,^19^ and people living with chronic headaches were involved throughout.^14,18,19^ In brief, the CHESS intervention consisted of 2 one-day group sessions 1 week apart (target group size 8–10), followed by a one-to-one nurse interview and telephone support. The group sessions focused on education and self-management to promote behavior change, healthy living, understanding chronic headache, and learning strategies to manage life despite headache. The one-to-one session and telephone follow-up supported drug management, lifestyle change, and goal setting. During goal setting, we used our classification interview approach to allow the nurses to provide disorder-specific advice including the use of migraine-specific acute treatments, use of preventive medications for migraine, and avoidance of medication overuse.

Sessions were co-led by a nurse and another registered allied health professional (nurse, health psychologist, physiotherapist, chiropractor, or occupational therapist) and just once by a research assistant. All facilitators attended 2 consecutive days of training covering the educational and self-management components. The nurses delivering the one-to-one sessions attended an additional training day to cover the classification interview and medication advice.

Study participants unable to attend the group they were originally allocated were offered 2 further groups to attend, if available. Quality control and assurance of the fidelity of intervention delivery was assessed by direct observation of sessions by members of the trial team with specific quality assurance feedback to facilitators as required. The protocol and results of the process evaluation have been published.^20,21^

Participants in the control group received a relaxation compact disk, something known to be a valued part of pain self-management programs.^22,23^ We also provided all participants, and their general practitioners, with the results of their headache classification interview and suggestions for appropriate drug management. This approach means we were able to isolate the effects of education and supportive self-management from the effects of headache classification and any resulting advice on drug management.

### Outcomes

Our primary outcome was headache-related quality of life measured using the Headache Impact Test (HIT)-6 at 12 months^24^ Secondary outcomes were the Chronic Headache Quality-of-Life Questionnaire v1.0; an adaption of the Migraine-Specific Quality-of-Life Questionnaire (v2.1) appropriate for our population, reported as role restrictions, limitations, and emotional impact of headaches^25^; headache days in the preceding 28 days; typical headache duration and severity in previous 28 days; EQ-5D-5L^26^; SF-12 v2 (version 2)^27^; Hospital Anxiety and Depression Scale (HADS)^28^; Pain Self-Efficacy Questionnaire (PSEQ)^29^; and Social activity: Social Integration Subscale of the Health Education Impact Questionnaire.^30^

We collected data on total headache days, average duration of headache, and headache severity from participants weekly for 6 months and then monthly, starting from the initial eligibility call to ensure we had prerandomization baseline data. Participants could report these outcomes either using a smartphone app or diary records.

At baseline, we collected basic demographic data, including ethnicity (White, Black or Black British, Asian or Asian British, Mixed, and other ethnic group), self-identified gender (male, female, other, and prefer not to say), and data on the troublesomeness of any other bodily pains.^31^ We collected patient-reported outcomes by post at 4, 8, and 12 months. If necessary, HIT-6, headache days, and EQ-5D-5L were collected by phone.

To show a difference of 2.0 on the HIT-6 at 12 months with an SD of 6.87, 90% power, an intracluster correlation of 0.01, and an average cluster size of 10 in the intervention group required data on 523 participants (253 control, 270 self-management; allocation ratio, 1:1.07).^32^ Assuming 95% of participants had chronic migraine or chronic tension–type headache and episodic migraine, and a 20% loss to follow-up, our minimum target recruitment was 689 (333 control, 356 self-management).^18^ Practicalities of delivering group interventions meant some over-run of this target was expected.

### Statistical Analysis

Our analyses followed the prespecified statistical analysis plan available in the Supplement (links.lww.com/WNL/C531). The primary approach was intention to treat on the complete case population. Data were reported in accordance with CONSORT guidelines.^33^ Analyses were conducted using the Stata 15 and R 4.0.3.

For primary and secondary analyses, treatment effects were estimated using linear mixed-effects models with partial clustering to account for clustering in the self-management arm. Analyses were adjusted for age, gender, and the baseline value stratification factors. Adjusted treatment effect estimates and associated 95% CI are presented for all analyses. All statistical tests were 2-sided at the 5% significance level. As per analysis plan, if the proportion of people with chronic tension–type headache only was <15%, main analyses would be on the population with chronic migraine or chronic tension–type headache and episodic migraine.

Drug use data for migraine (except Botox and calcitonin gene–related peptide monoclonal antibodies) reported in participant questionnaires were converted to amounts taken over the previous 28 days and then converted to defined daily doses (DDD).^34^ Opioids were standardized to DDD of codeine using a morphine equivalence table (personal communication I-WOTCH study team). Results are presented for drug group and type (acute/preventive) see eTables 1–8, links.lww.com/WNL/C531. Our drug use data were not suitable for parametric analysis. We therefore reported proportion using medication and a nonparametric Wilcoxon rank-sum test in those using the medications.

We predefined minimal adherence to the intervention as the participant attending day 1 of the intervention plus the one-to-one session with the nurse and full adherence as the participant attending the entire intervention. We performed complier averaged causal effect (CACE) analyses for both levels of adherence for the primary outcome only to estimate the difference between observed compliers (intervention) and potential compliers (control).^35^

We performed prespecified subgroup analyses to examine whether baseline anxiety (HADS anxiety subscale scores ≥11), depression (HADS depression subscale ≥11), and severity (HIT-6 ≤64 and >64) moderated treatment effect for primary outcome only.^36,37^

Headache days, headache duration, and severity were reported by participants at multiple time points. To account for the within-subject dependency, each outcome was analyzed using a mixed-effects model to estimate the treatment effect over time with random effects at the participant level. The models were adjusted for the same variables as in the primary analyses (fixed effects).

We presented the primary outcome separately for the following: whole population, those with chronic migraine, or those with chronic tension–type headache and episodic migraine, and those with or without medication overuse. The small number with only chronic tension–type headache precluded presenting data on these individuals separately.

We performed 2 sensitivity analyses: (1) excluding participants who were included in the process evaluation interviews and^20,21^ (2) excluding those participants who reported <15 headache days in the previous 28 days in the baseline questionnaire.

Adverse events (AEs) and serious adverse events (SAEs) were summarized as frequencies and percentages (%). If possible, the 2 arms were compared using either the χ^2^ test or Fisher exact test. Adjusted analyses were not performed for any of these data.

We performed a prospective within-trial economic evaluation from the perspective of the UK National Health Service and Personal Social Services.^38^ We conducted a cost-utility analysis, expressed in incremental cost per quality-adjusted life year (QALY) gained. We obtained unit costs (2019 £) (converted into 2019 USD using gross domestic product deflator index values and purchasing power parity conversion rates produced by the International Monetary Fund) from primary and secondary sources in accordance with national guidelines and attached them to every item of resource use. QALY profiles were calculated for each participant using health utility scores generated from the EQ-5D-5L and assuming linear interpolation between baseline and follow-up health utility scores. We conducted a bivariate generalized linear mixed-effects regression of costs and QALYs, with multiple imputation of missing data, to estimate the incremental cost per QALY gained for the CHESS intervention compared with usual care. Further details of the economic evaluation are provided in the Supplement (eAppendix 1, links.lww.com/WNL/C531).

### Standard Protocol Approvals, Registrations, and Patient Consents

North West—Greater Manchester East Research Ethics Committee approved the trial (REC REF: 16/NW/0890). Participants provided written consent. The trial was registered on the International Standard Randomized Controlled Trial Number registry, ISRCTN79708100. The trial protocol is available in the Supplement (links.lww.com/WNL/C531).

### Data Availability

Individual participant data and a data dictionary will be available, subject to a data sharing agreement, for further prespecified analyses on request through Warwick Clinical Trials Unit (wctudataaccess@warwick.ac.uk), following publication of the funder report.

## Results

**Figure 1:**
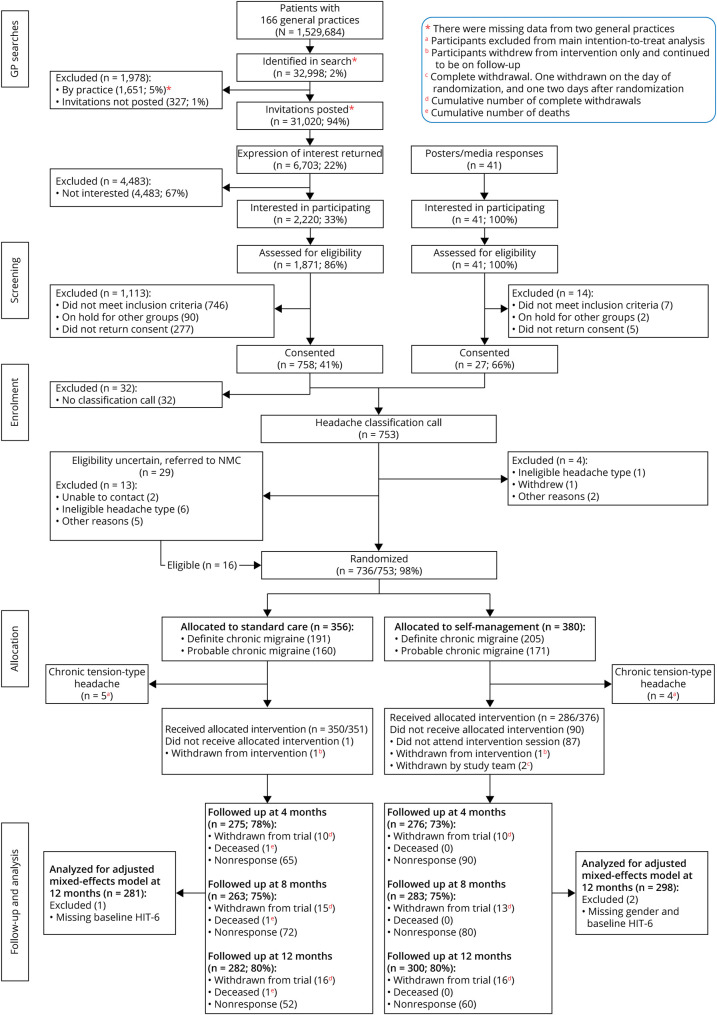
CONSORT Chart

We approached 31,020 people from 166 general practices across London and the Midlands (combined list size = 1,529,684); 2,220 expressed an interest in the trial and 41 people self-referred. Of them, 1,912 (85%) were contactable, and 1,159 (61%) of them were eligible. We randomized 736 (64%) of these people between 24 April 2017 and 31 March 2019 (Figure 1, eTables 9 and 10, links.lww.com/WNL/C531). The median time between confirmation of eligibility and baseline questionnaire completion was 8 days (IQR, 5–13).

Because of the nature of the group intervention (fixed dates and times), not everyone who completed eligibility assessment could access the intervention; thus, it was not possible to randomize all those eligible for the trial (Figure 1).

Nine participants (1%) had chronic tension–type headache, so our main analyses were on the remaining 727 with chronic migraine or chronic tension–type headache and episodic migraine. Of them, we classified 396/727 (54%) as having chronic migraine; 407/727 (56%) also had medication overuse headache (Table 1, eFigure 1 and eTable 11, links.lww.com/WNL/C531). Participants were mainly female (604/727, 83%) with a mean age of 48 (SD, 15) years; 131/727 (18%) identified their ethnicity as Asian, Black, or mixed. The median number of headache days per month at baseline was 16 (IQR, 11–20); 274/727 (38%) reported <15 days of headache in previous 4 weeks. The DDD of acute treatments for those in the self-management arm in the preceding 4 weeks was 12 (IQR, 5.3–25; Table 2), which was comparable with those in the standard care (median DDD, 14; IQR, 6.6–28). A third 235/727 (32%) had used prophylactic medications (standard care, median DDD, 14; IQR, 5.3–32 vs self-management, median, 14; IQR, 6.9–28; Table 2). The mean HIT-6 score at baseline was 64.5 (SD, 5.5) (Table 1, eTable 12). More than half of participants (382/727; 53%) had probable anxiety (HADS anxiety score ≥11) and 1 in 5 (159/727; 22%) of participants had probable depression (HADS depression score ≥11). Those with chronic migraine were more severely affected by their headaches than those with chronic tension–type headache and episodic migraine (eTable 12). Other chronic pains were common; 375/727 (52%) participants had at least moderately troublesome neck pain and 277/727 (38%) participants had at least moderately troublesome back pain (eTables 13 and 14). The 2 different treatment groups were well matched on baseline characteristics (Table 1, eTables 11 and 12).

**Table 1:**
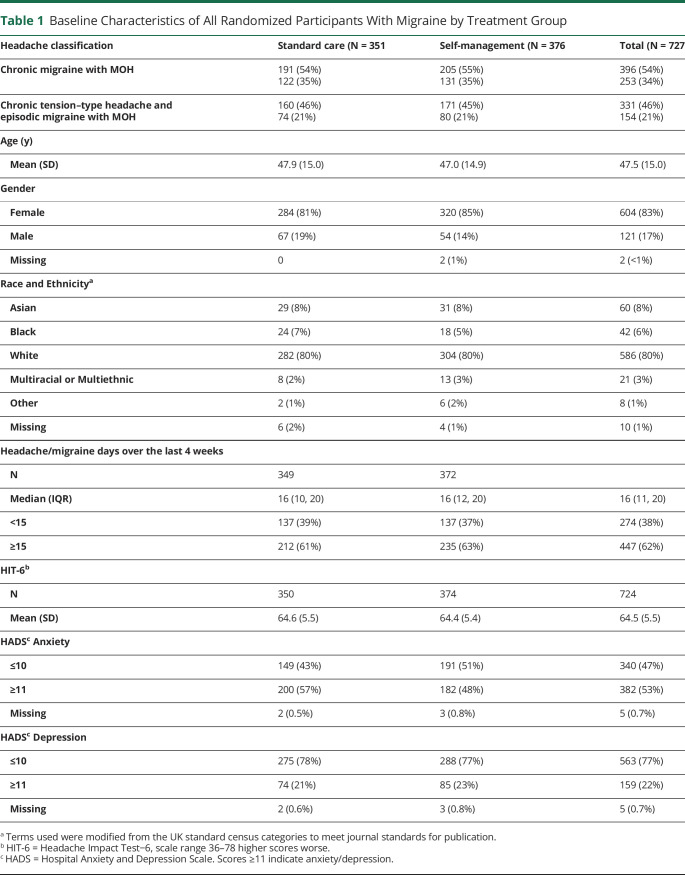
Baseline Characteristics of All Randomized Participants With Migraine by Treatment Group

**Table 2:**
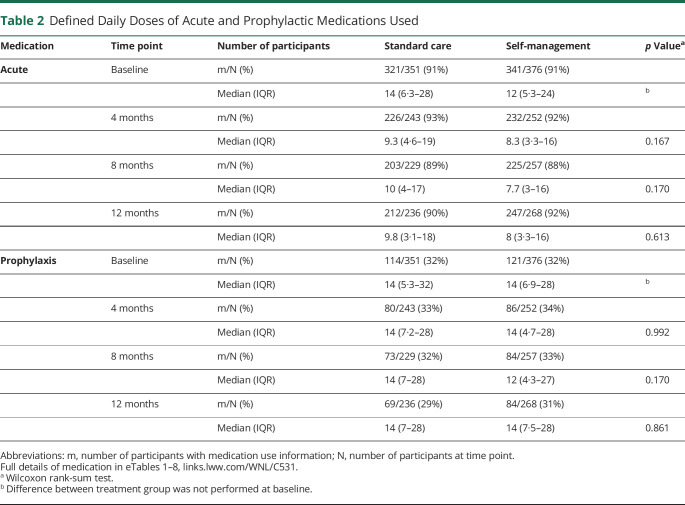
Defined Daily Doses of Acute and Prophylactic Medications Used

We held 42 self-management groups across 35 locations; 286/376 (76%) participants attended the first session, with a median group size of 6.5 (IQR 5–9), 259/376 (69%) achieved the predetermined minimum adherence (day 1 and one-to-one sessions), and 216/376 (58%) achieved full adherence to the program (eTable 15, links.lww.com/WNL/C531). The median time from randomization to the first treatment session was 15 days (IQR, 11–23).

We obtained analyzable primary outcome data from 586 participants with chronic migraine or chronic tension–type headache and episodic migraine (81%) at 12 months. There was no between-group difference in HIT-6 (adjusted mean difference, −0.3; 95% CI, −1.23 to 0.67; *p* = 0.56, (Table 3). At 4 months only, there was a difference favoring our self-management program (adjusted mean difference, −1.0; 95% CI, −1.91 to −0.006; *p* = 0.049). Results of our CACE analyses were not materially different (Table 3).

**Table 3:**
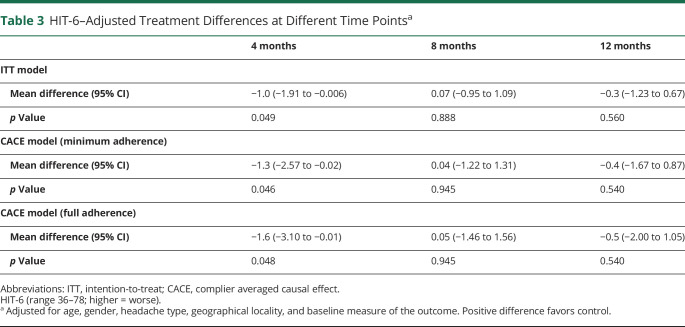
HIT-6–Adjusted Treatment Differences at Different Time Points^a^

Smartphone app/diary data were poorly completed, median completion rate approximately 44%, making imputation inappropriate. The between-group difference over 12 months for the number of headache days was 0.2 (95% CI, −0.11 to 0.46; *p* = 0.234); for the duration of headache, the estimated difference was 0.4 (95% CI, −0.47 to 1.28; *p* = 0.361), and for headache severity, the estimated difference was 0.2 (95% CI, −0.08 to 0.46; *p* = 0.163). (eTable 16, links.lww.com/WNL/C531).

**Figure 2:**
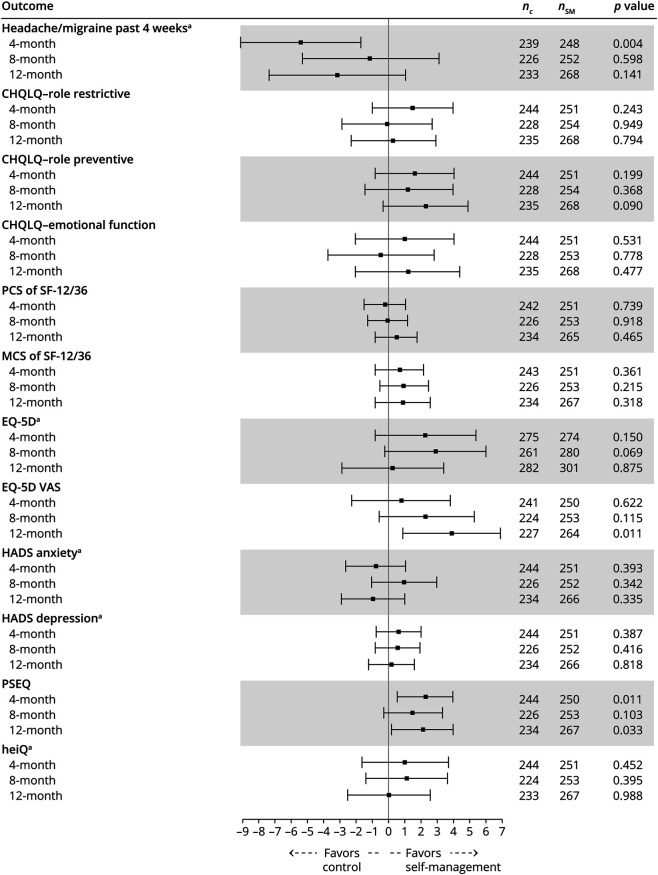
Treatment Differences and 95% CI for Secondary Outcomes, Adjusted for Age, Gender, Baseline Value of the Dependent Variable, Headache Type, and Geographical Locality at 4-, 8- and 12-Month Follow-ups Abbreviations: nC, number of participants from standard care; nSM, number of participants from self-management. Estimates and 95% CI rescaled to range from 0 to 100 for graphical representation purposes only. To obtain the estimated difference and its 95% CI in its original scale, the value from graph is multiplied by maximum value/100. For example, the estimated difference for HADS Anxiety at 4-month FU was −0.801 × 21/100 = −0.16821). See also eTables 17–19 (links.lww.com/WNL/C531). HADS = Hospital Anxiety and Depression Scale.

There were few differences in our secondary outcomes (Figure 2, eTables 17–19 , links.lww.com/WNL/C531). People in the self-management group reported 1.5 (95% CI, 0.48–2.56; *p* = 0.004) more headache days over the previous 4 weeks at 4 months of follow-up, but not at 8 and 12 months. There were benefits in improving PSEQ at 4 and 12 months but not at 8 months. The overall numbers using acute and prophylactic drugs, and amounts used, were unchanged over time with no between-group differences (Table 2). There were a few statistical differences in the use of individual drug groups over time (eTables 3–8). There were no differences in proportions using acute medications ≥10 or ≥15 days in previous 28 days at any follow-up, indicating no effect on medication overuse (eTables 20–22). Second-line prophylactic drugs (Botox & CGRP monoclonal antibodies) were used; 4 received Botox injection (n = 2 each arm), 2 from the self-management arm were prescribed erenumab, and 1 received both botox and erenumab (results not shown). We found no evidence of subgroup effects in our preplanned analyses for anxiety, depression, and headache severity (Table 4). The effect on HIT-6 at 12 months in those with chronic migraine was −0.7 (95% CI −1.97 to 0.65, *p* = 0.325) and in those with chronic tension–type headache and episodic migraine was −0.1 (95% CI −1.46 to 1.35, *p* = 0.943) (eTable 23). For those with medication overuse headache, it was −0.03 (95% CI −1.31 to 1.26, *p* = 0.967); for those without medication overuse, it was −0.4 (95% CI −1.85 to 0.95), and for those with ≥15 days of headaches in their baseline questionnaire, the difference was −0.2 (95% CI −1.45 to 0.97; *p* = 0.696) (eTables 23 and 24). For the whole population, including those with chronic tension–type headache only, it was −0.3 (95% CI −1·22 to 0.66; *p* = 0.555); (eTable 25). For all our analyses, the intracluster correlation coefficient in the intervention arm was <0.001.

**Table 4:**
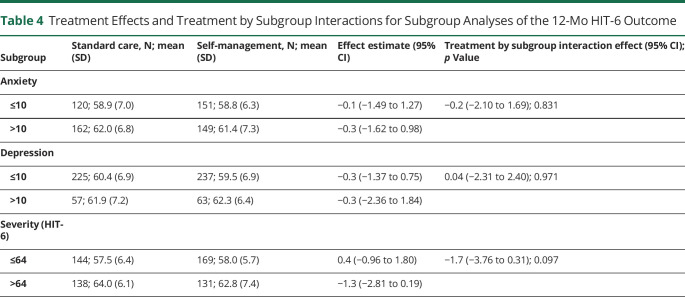
Treatment Effects and Treatment by Subgroup Interactions for Subgroup Analyses of the 12-Mo HIT-6 Outcome

There were 7 AEs, 1 in the standard care arm and 6 in the self-management arm. There was 1 SAE; a participant in the standard care arm died of an unrelated cause (eTable 26, links.lww.com/WNL/C531).

**Figure 3:**
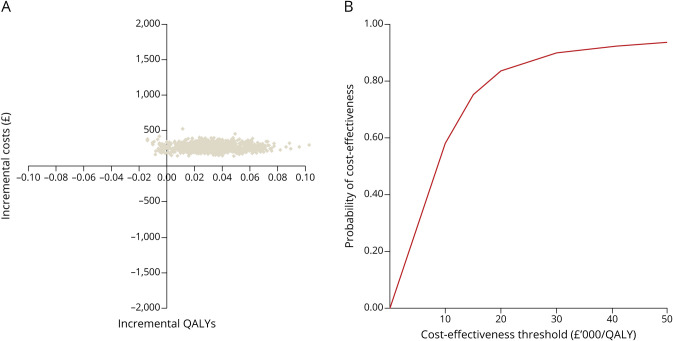
Cost-effectiveness Plane Displaying Incremental Costs and QALYs and Cost-effectiveness Acceptability Curves and Probability Estimate of the Intervention Compared With Usual Care at the Specified Willingness-to-Pay Thresholds (A) The graph shows the cost-effectiveness plane displaying 1,000 base-case ICERs simulated from the joint distribution of incremental costs and incremental QALYs. (B) Graph represents cost-effectiveness acceptability curves and gives a probability estimate of the CHESS intervention being cost-effective compared with usual care at the specified willingness-to-pay thresholds. Abbreviations: CHESS = Chronic Headache Education and Self-management Study; ICER = incremental cost-effectiveness ratio; QALY = quality-adjusted life year.

The CHESS intervention generated incremental adjusted costs of £268 (95% CI £176–£377) [USD383 (95%CI USD252–USD539)] and incremental adjusted QALYs of 0.031 (95% CI −0.005 to 0.063). The incremental cost-effectiveness ratio was £8,617 (USD12,322) per QALY gained. The incremental net monetary benefit was £354 (95% CI −£375 to £1,084) [USD506 (95% CI −USD536 to USD1,550)] with probability that the intervention is cost-effective, approaching 0.83 if the cost-effectiveness threshold is £20,000 (USD28,600) per QALY gained (Figure 3 and eAppendix 1, links.lww.com/WNL/C531). This study provides Class III evidence that a brief group education and self-management program does not increase the probability of improvement in headache-related quality of life in patients with chronic migraine.

## Discussion

There was no indication that the CHESS intervention had any important beneficial effects on clinically relevant outcomes. Only at 4 months was there an indication, on balance, that there was beneficial effect on the HIT-6, −1.0 (95% CI −1.91 to −0.006); *p* = 0.049). This was small, just half of our target (worthwhile) difference of 2.0. This needs to be set against participants in the intervention arm reporting 1.5 (95% CI, 0.48–2.56; *p* = 0.004) more headache/migraine days in the previous 28 days than control participants at 4 months. We did not find any evidence of any benefit in any of our preplanned subgroup and sensitivity analyses. We had ample statistical power to identify any clinically important between-group differences because we exceeded our target sample size and clustering effects in the intervention arm were negligible. The limits of the 95% CI, for the primary outcome, do not include our target difference; effectively excluding any possibility, the CHESS intervention has a worthwhile effect on HIT-6. These conclusions apply equally to the overall analysis including all 736 randomized participants.

In our secondary outcomes, only for pain self-efficacy was there a benefit from treatment, observed at 4 and 12 months but not at 8 months. This may just be a chance finding because of multiple comparisons. However, it might indicate that our intervention does improve self-efficacy, one of our key intervention targets, but that this does not translate into a measured patient benefit.

During trial design, the most appropriate measure for a population that may not have been given a headache diagnosis was the HIT-6.^39^ The CHQLQ better reflects the concerns of people living with migraine and has good measurement properties in our population of interest.^40^ It was included here pending completion of validation. However, this more patient-focused measure also does not show any benefit at any time point in any of its three dimensions.

A quarter of people in the intervention group did not attend any treatment sessions. This was despite participants confirming they were available on proposed dates before randomization. Nonattendance is common in trials of group interventions for chronic pain, for example, 17% and 11% in 2 similar studies.^41,42^ The unpredictable nature of headache disorders might explain the higher nonattendance rate in this study. Nevertheless, the minimal adherence rate in the CHESS of 69% compares favorably with minimal adherence rates of 70% and 63%, respectively, in these previous studies.^41,42^ Our CACE analyses provided very similar estimates of effect sizes to the ITT analyses, indicating that better adherence to the intervention is unlikely to increase benefit from the intervention.

Although diaries were poorly completed, the findings were consistent with main results. Defining the population of interest is important for trials of interventions of headache disorders. It is also important that criteria developed for evaluating drug treatments for pain are not inappropriately applied to studies on nonpharmacological treatments.^43^ This study was originally designed to study a population meeting an epidemiologic definition of chronic headache. This maps onto the point in the care pathway, in primary care, where a general (family) practitioner might refer people to such a treatment program, which includes a headache classification, following a single consultation rather than following completion of a headache diary. Our main results are describing the effect on the majority population classified as having chronic migraine or chronic tension–type headache and episodic migraine after a single nurse interview. While the interview has been validated, the population may not be the same as those diagnosed with chronic migraine by a headache specialist.^15^ It is reassuring that findings were not materially different for those with chronic migraine or chronic tension–type headache and episodic migraine; this gives some reassurance that findings apply to all those with “chronic migraine.” Although the presence of chronic headache was an entry criterion for this study, and the median delay between study entry assessment and completion of the baseline questionnaire was just 8 days, only 62% reported that they had had headaches on 15 or more days in the preceding 3 months in their baseline questionnaire. This may be partly because of the known short-term variability in headache days and possibly some response shift in questionnaire completion.^44,45^ Nevertheless, it is possible we included some participants who did not meet diagnostic criteria for chronic migraine or chronic tension–type headache and episodic migraine. The population recruited was, however, the population that would have been offered the CHESS intervention if it was shown to be successful: meaning our findings are directly applicable to clinical practice in primary care. The treatment effect was not materially different from the overall estimate in those reporting ≥15 headache days in the previous month; this suggests our findings are applicable to those formally diagnosed with chronic migraine or chronic tension–type headache and episodic migraine.

The CHESS intervention in the absence of a clinical effect seems to generate additional QALYs and has a high probability of cost-effectiveness given UK cost-effectiveness thresholds. The EQ-5D-5L might be measuring nonspecific effects not captured by the HIT-6 or it might be that the early effect on headache-related disability has had a larger proportional effect in the area under the curve analysis.

The control intervention was more than just usual care; the results of the classification interview were fed back to participants and their GPs, which might have reduced any potential effect size from the CHESS intervention if people in the control group used medication more appropriately in light of our feedback. However, the absence of any differences over time in either group in the use of prophylactic medications make it unlikely that improved diagnosis in the control group affected our findings.

The trial found no evidence of any clinically relevant benefit from the CHESS intervention across multiple outcomes, at multiple time points, or in any sensitivity or subgroup analyses. It clearly demonstrates the intervention tested here is ineffective and not detrimental. This is surprising because the CHESS intervention targeted the key modifiable psychological variables known to be predictive of poor prognosis in chronic headache disorders, had a solid theoretical underpinning, intervention fidelity was high, and it was well regarded by participants and facilitators.^21^ Only 3/21 studies (N = 183) in a 2019 Cochrane review of psychological therapies for the prevention of migraine were predominately of people likely to have chronic migraine.^10,46-48^ Overall, these studies and our trial do not indicate that behavioral/educational interventions have any meaningful effect on clinical outcomes for people with chronic migraine. A search of trial registries (June 2022) identified 2 trials of behavioral interventions for chronic migraine in progress; a mindfulness intervention for chronic migraine, ClinicalTrials.gov Identifier: NCT03671681, and a health education program for the prevention of chronic migraine NCT04788667.

In conclusion, our data effectively exclude the possibility that this short intervention is effective for the treatment of chronic migraine or chronic tension–type headache and episodic migraine. There remains a need to identify more effective treatments for people living with, the sometimes disabling, symptoms of chronic migraine or chronic tension–type headache and episodic migraine.

## Glossary

AE: adverse events
CACE: Complier Averaged Causal Effect
CHESS: Chronic Headache Education and Self-management Study
DDD: defined daily dose
HADS: Hospital Anxiety and Depression Scale
HIT-6: Headache Impact Test
ICHD-3: *International Classification of Headache Disorders, 3rd edition*
PSEQ: Pain Self-Efficacy Questionnaire
SAE: serious AE
